# Enhancing the endo-activity of the thermophilic chitinase to yield chitooligosaccharides with high degrees of polymerization

**DOI:** 10.1186/s40643-024-00735-x

**Published:** 2024-03-07

**Authors:** Feifei Guan, Xiaoqian Tian, Ruohan Zhang, Yan Zhang, Ningfeng Wu, Jilu Sun, Honglian Zhang, Tao Tu, Huiying Luo, Bin Yao, Jian Tian, Huoqing Huang

**Affiliations:** 1 Institute of Animal Science, Chinese Academy of Agricultural Sciences, Beijing, 100193 China; 2 Biotechnology Research Institute, Chinese Academy of Agricultural Sciences, Beijing, 100081 China; 3 College of Food Science and Technology, Hebei Agricultural University, Hebei Baoding, 071000 China

**Keywords:** Chitin, Chitinase, Endo-activity, Mutants, Functional oligosaccharides

## Abstract

Thermophilic endo-chitinases are essential for production of highly polymerized chitooligosaccharides, which are advantageous for plant immunity, animal nutrition and health. However, thermophilic endo-chitinases are scarce and the transformation from exo- to endo-activity of chitinases is still a challenging problem. In this study, to enhance the endo-activity of the thermophilic chitinase Chi304, we proposed two approaches for rational design based on comprehensive structural and evolutionary analyses. Four effective single-point mutants were identified among 28 designed mutations. The ratio of (GlcNAc)_3_ to (GlcNAc)_2_ quantity (DP_3/2_) in the hydrolysates of the four single-point mutants undertaking colloidal chitin degradation were 1.89, 1.65, 1.24, and 1.38 times that of Chi304, respectively. When combining to double-point mutants, the DP_3/2_ proportions produced by F79A/W140R, F79A/M264L, F79A/W272R, and M264L/W272R were 2.06, 1.67, 1.82, and 1.86 times that of Chi304 and all four double-point mutants exhibited enhanced endo-activity. When applied to produce chitooligosaccharides (DP ≥ 3), F79A/W140R accumulated the most (GlcNAc)_4_, while M264L/W272R was the best to produce (GlcNAc)_3_, which was 2.28 times that of Chi304. The two mutants had exposed shallower substrate-binding pockets and stronger binding abilities to shape the substrate. Overall, this research offers a practical approach to altering the cutting pattern of a chitinase to generate functional chitooligosaccharides.

**Graphical abstract:**

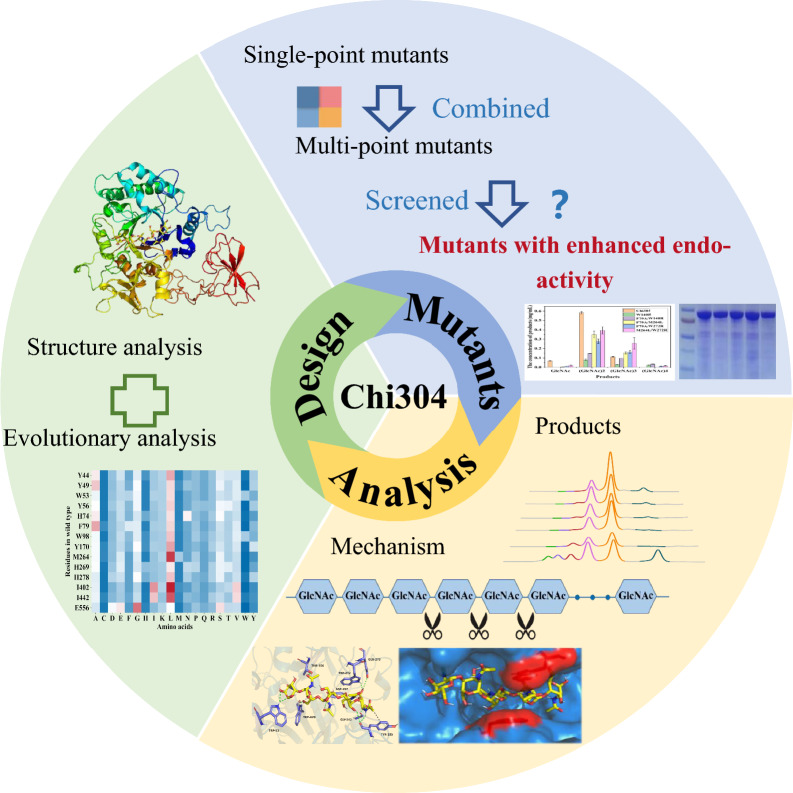

**Supplementary Information:**

The online version contains supplementary material available at 10.1186/s40643-024-00735-x.

## Introduction

Chitin is a natural polymeric polysaccharide polymerized by *N*-acetylglucosamine through β-1-4 glucoside bonding and widely distributed in nature as the shell material of arthropods, etc. Chitin is among the most abundant polysaccharides on Earth, second only to cellulose (Tsurkan et al. 2021). The degradation products of chitin are mostly chitin oligosaccharides with degrees of polymerization (DP) of 2–20. Compared to products with low degrees of polymerization, highly polymerized products (DP ≥ 3) have greater value in pharmaceutical (Liaqat and Eltem 2018), plant immunity (Hayafune et al. 2014; Liu et al. 2012; Yamada et al. 1993), animal nutrition, and health (Deng et al. 2020; Zheng et al. 2018). Chitinase is a key hydrolase in the biological degradation of chitin. Chitooligosaccharides with high degrees of polymerization can be obtained using endo-chitinases. Although many chitinases are now being reported and exhibited remarkable performance in catalytic efficiency (Madhuprakash et al. 2015; Su et al. 2021) or thermostability (Kozome et al. 2022; Xu et al. 2020), etc., the transition of exo- and endo-activity of chitinases remains mysterious and unattended.

Up to now, based on the structural analyses, there are some preliminary studies on the molecular modification and mechanism of endo-chitinase, but it is still a difficult challenge. As reported, the binding site and binding mode between chitinase and substrate can differ, altering chitinase function. The exo-chitinases ChiA and ChiB have deep, tube-shaped substrate-binding pockets, whereas the endo-chitinase ChiC has shallow substrate-binding channels (Baban et al. 2010) and must temporarily restructure the loops that comprise the roof of the substrate-binding groove (Sikorski et al. 2006). On the other hand, studies on the cleavage mechanism and conversion of chitinase endo-activity showed that aromatic amino acids in the binding pocket also play an important role. For example, an aromatic amino acid residue (Trp97) found in the substrate-binding cleft of SmChiB leads it to hydrolyze partially N-acetylated chitosan processively, while NtChiV, whose amino acid sequence is similar to that of the SmChiB, catalyzes non-processive hydrolysis of the same substrate due to substitution of the aromatic amino acid residue with an aliphatic residue (Ohnuma et al. 2011). For the typical exo-chitinases ChiA (Zakariassen et al. 2009) and ChiB (Horn et al. 2006), when aromatic amino acid residues (W167 and W275 in ChiA, W97 and W220 in ChiB) near the active center or in the substrate-binding pocket were mutated to alanine, endo-activity increased to varying degrees. However, based on the structure–function relationship, the effects of amino acids with different side chains or properties (except for A) around the substrate-binding cleft on the cleavage mode of chitinase have not been studied and their potential mechanisms remain unclear and challenging.

In addition, some natural endo-chitinases have been cloned from the genome of microorganisms or plants, and most of them belong to the GH19 family. For example, SaChiB from *Streptomyces alfalfae* ACCC 40021 (Lv et al. 2021), and chitinases found in the rice genome, OsChia1cΔChBD and OsChib1a, appear to employ a non-processive endo-mode of action (Sasaki et al. 2003, 2006). Chitinases from the GH18 and GH19 families have varied amino acid sequences, structures and catalytic reaction mechanisms (Adrangi and Faramarzi 2013; Berini et al. 2018; Li and Greene 2010). Chitinases from GH18 family possess the typical TIM-barrel and a conserved DXDXE motif, while those from family 19 have more α helices (Li and Greene 2010). During the evolution of these endo-chitinases, there may be some conserved key amino acid residues or features contributing to the endo-cleavage. In recent years, the modification of enzymes based on sequence-function relationship is gradually mature and successful (Freschlin et al. 2022; Song et al. 2021). Gathering the sequence-function data to guide the modification of target enzymes further improves the possibility of obtaining positive mutants and thus identified the conserved amino acids (Weinstein et al. 2020). However, from the perspective of the whole protein sequence, there are few studies involving finding key sites related to the cutting mode of chitinases or other enzymes.

The thermophilic chitinase Chi304 (GenBank accession number: MW446948) derived from marine metagenome mainly exerted exo-activity, with maximum activity at 85 °C and pH 9.0 (Zhang et al. 2022). The exo-activity of Chi304 generally involves the successive removal of dimers from long chitin chains. In this study, we hypothesized that the conserved amino acids in natural endo-chitinase, as well as those located at or near the substrate binding pocket, have an effect on the conversion from exo- to endo-activity of chitinases. Through structural and evolutionary analysis, we sought to boost endo-activity of Chi304, in order to generate highly polymerized products (DP ≥ 3) and analyze the crucial amino acids affecting substrate cleavage mode and the conversion mechanisms associated with endo- and exo-activities.

## Materials and methods

### General

This study was conducted based on previous research. For information about the crude chitin powder, GlcNAc, (GlcNAc)_2–6_, and the preparation of colloidal chitin, please refer to the work of Zhang (2022).

### Homology modeling of Chi304 and its mutants

Homology models of Chi304 and its mutants (F79A/W140R and M264L/W272R) were constructed using two chitinase structures as templates. Sequence analysis identified homologs of Chi304 sequences using the SWISS-MODEL (https://swissmodel.expasy.org/) server, and the first two templates, SmChiAB-FYSFV (SMTL ID: 5zl9.1) and ChiA74 (SMTL ID: 6bt9.1), were selected for model construction. Discovery Studio (2017) software (Accelrys, San Diego, CA, USA) was used for homology model construction according to the manufacturer’s instructions.

### Protein–ligand interaction study

The ligand, penta-*N*-acetylchitopentaose, was drawn using ChemBioDraw Ultra 14.0, and energy minimization was conducted using ChemBio3D Ultra 14.0. The binding sites of Chi304 were conserved and were used as search queries in the Conserved Domains Database (CDD) of the National Center for Biotechnology Information (NCBI; https://www.ncbi.nlm.nih.gov/Structure/cdd/wrpsb.cgi). AutoDock4 Vina (Morris et al. 2009) was used to conduct docking analysis between Chi304 and penta-N-acetylchitopentaose. A total of eight docking conformations were calculated, and the most reasonable and stable conformation was selected for subsequent analysis. Interactions between the macromolecular protein and the ligand were analyzed using the Protein–Ligand Interaction Profiler (https://plip-tool.biotec.tu-dresden.de/plip-web/plip/index) (Adasme et al. 2021) and verified with PyMOL 4.6.

### Evolutionary analysis of GH19-family chitinases

The protein sequence of endo-chitinase OsChia1c (GH19 family) was obtained from NCBI (https://www.ncbi.nlm.nih.gov/). Then, the Basic Local Alignment Search Tool (BLAST) (Altschul et al. 1997) was employed, searching the nr database with criteria of *e*-value < 0.0001 and alignment match percent ≥ 70%. A total of 706 sequences were downloaded and aligned with Chi304 using MUSCLE software. The position-specific amino acid possibility (PSAP) at each position of Chi304 was calculated using the Parepro program (Tian et al. 2007).

### Site-directed mutagenesis

Single- and double-point mutants were constructed using a two-step polymerase chain reaction mutagenesis strategy (Kirsch and Joly 1998). The primers used to generate the mutants were designed using Oligo software ver. 7.0 and are listed in Table S1. First, a DNA fragment containing the mutation site was amplified using a reaction mixture of 25 μL 2 × Phanta Max Buffer, 3 μL each primer (10 μM), 1 μL dNTPs (10 mM), 1 μL pET30a ( +)-*chi304*, 1 μL Phanta Max Super-Fidelity DNA Polymerase (Vazyme, Nanjing, China), and 16 μL ddH_2_O. The reaction conditions were 94 °C for 3 min; 32 cycles at 94 °C for 30 s, 56 °C for 30 s, and 72 °C for 45 s; and final elongation at 72 °C for 10 min. DNA fragments were gel-purified using the AxyPrep DNA Gel Extraction Kit (Corning, Hangzhou, China). Then, 500 ng of the product was used as a megaprimer in a reaction with 1 μL pET30a ( +)-*chi304* plasmid as the template, 25 μL 2 × Phanta Max Buffer, 1 μL dNTPs (10 mM), 1 μL Phanta Max Super-Fidelity DNA Polymerase, and ddH_2_O to a final volume of 50 μL. The following amplification program was used: 94 °C for 3 min, 32 cycles at 94 °C for 30 s, 72 °C for 140 s, and a final elongation step at 72 °C for 10 min. The mixed products were digested using *Dpn*I (NEB, Ipswich, MA, USA) at 37 °C for 1 h and cleaned using a Universal DNA Purification Kit (Tiangen Corp. Beijing, China). The purified fragments were transferred into *Escherichia coli* TOP10, and clones were selected for sequencing verification.

### Protein expression, purification, and enzymatic activity measurement

The constructed mutant plasmids were transformed into *E*. *coli* BL21 (ED3) and positive clones were selected. The induction, expression, and purification of the mutants were conducted according to the procedure reported for Chi304 (Zhang et al. 2022). The enzymatic activity of Chi304 and its mutants were measured using 3,5-dinitrosalicylic acid solution against colloidal chitin at 85 °C and pH 9.0 (glycine–NaOH, 100 mM), as described previously (Zhang et al. 2022).

### Detection of hydrolysis product composition from Chi304 and mutants

With colloidal chitin as the substrate, chitinase Chi304 and its mutants were diluted to equal enzymatic activity of 0.25 U, and for (GlcNAc)_6_ as the substrate, enzymatic activity was set to 0.05 U. Then, 250 μL of substrate and 500 μL of reaction buffer (glycine–NaOH, 0.05 M, pH 9.0) were heated to 85 °C for 2 min, after which 250 μL of enzyme solution was added for 30 min of reaction. The reaction was terminated with the immediate addition of 1 mL chloroform. Next, the reaction mixture was centrifuged at 13,400 × *g* for 1 min, and the supernatant was filtered through a 0.22-μm aqueous membrane for detection. High-performance liquid chromatography (HPLC) conditions were as follows: column, Shodex suger KS-802; detector, Shimadu RID-20A; separation phase, HPLC water; flow rate, 0.6 mL/min; detection time, 30 min; column temperature, 65 °C; and injection volume, 20 μL. The concentration of each component of the mixed standard (GlcNAc)_1–6_ was 2 mg/mL.

### Monitoring the time course of oligosaccharide hydrolysis by chitinase

The concentration of chitinase Chi304 was diluted consistent with the mutant to 0.002 U for dehydrolysis of (GlcNAc)_6_. The reaction conditions were as described above, with reaction times of 0, 2, 5, 10, 30, 60, and 120 min. After termination of the reaction, the products were assayed using HPLC.

## Results

### W140 and W272 were predicted to be key sites in structural analysis

**Fig. 1:**
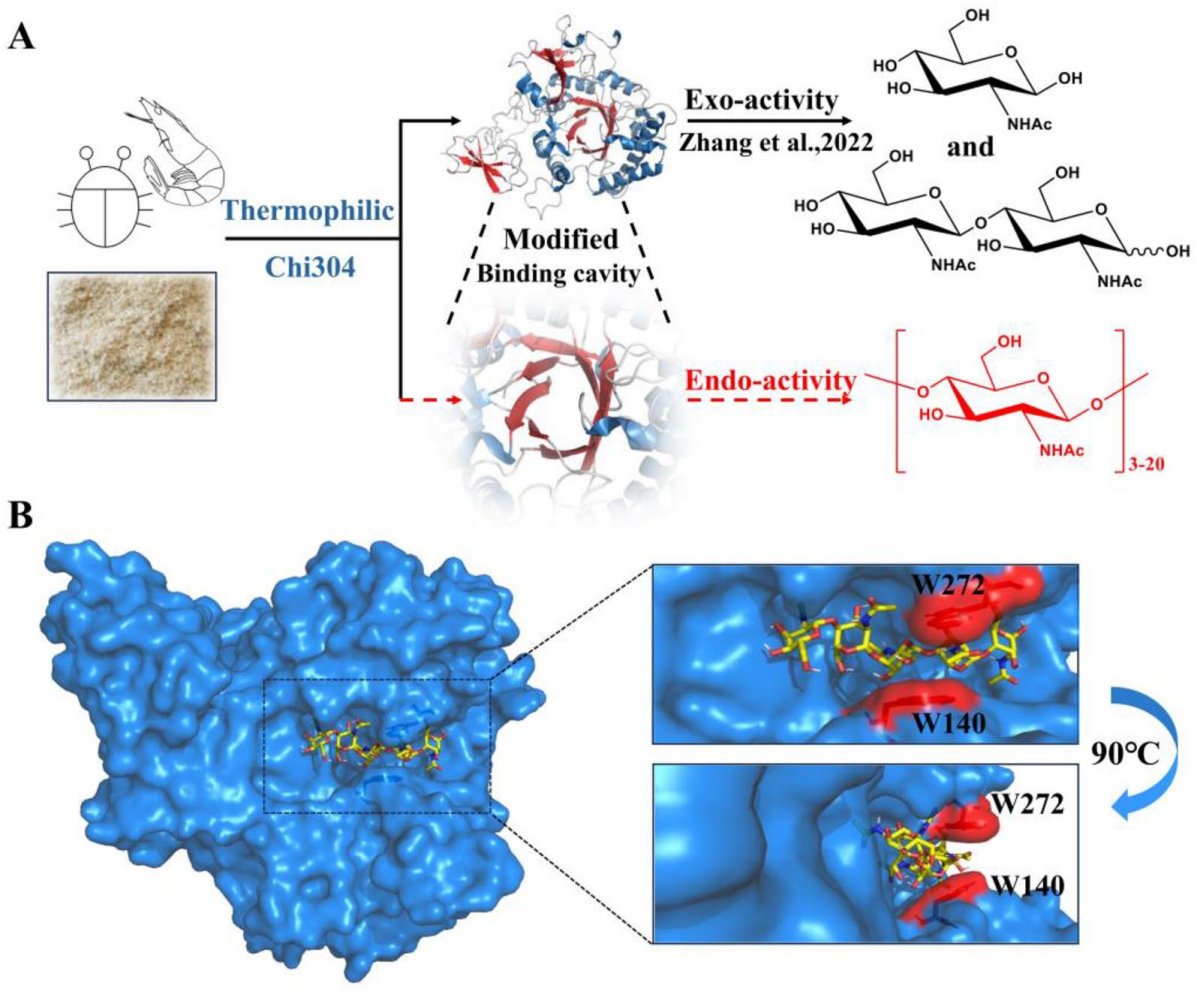
Overview of the study (**A**) and docking analysis between the homologous structure of Chi304 and substrate penta-*N*-acetylchitopentaose (**B**)

Endo-chitinase is a key hydrolase in the biological degradation of chitin to produce chitin oligosaccharides with DP ≥ 3. Chi304, a thermophilic chitinase mined directly from a marine metagenome, is a member of the glycoside hydrolase 18 family. Previous research has demonstrated that Chi304 is capable of efficiently breaking down colloidal chitin, crude chitin powder to produce diacetyl-chitobiose and *N*-acetylglucosamine, demonstrated mainly exo-activity (Zhang et al. 2022) (Fig. 1A). In order to modified Chi304 for endo-activity, the key amino acid residues located at or near the catalytic core and substrate-binding cavity were identified. The results of showed that Chi304 has a deep substrate-binding channel. Tryptophans at positions 140 and 272 were predictably critical, as the benzene rings of the side chain likely help to narrow the deep cleft, supporting the exo mode of binding to substrates (Baban et al. 2010) (Fig. 1B). To further investigate the functions of W140 and W272 in the binding cavity of Chi304, 14 mutants with different side chains and charge properties were designed, as listed in Table 1.

**Table 1:** Mutants designed for W140 and W272 through structural analysis

No.	Site	Mutant AA
1	Non-polar	A
2		F
3		G
4		L
5	Polar	Q (uncharged)
6		R (positively charged)
7		E (negatively charged)

### Accumulation of highly polymerized products with diminished exo-activity and enhanced endo-activity in W140R and W272R

**Fig. 2:**
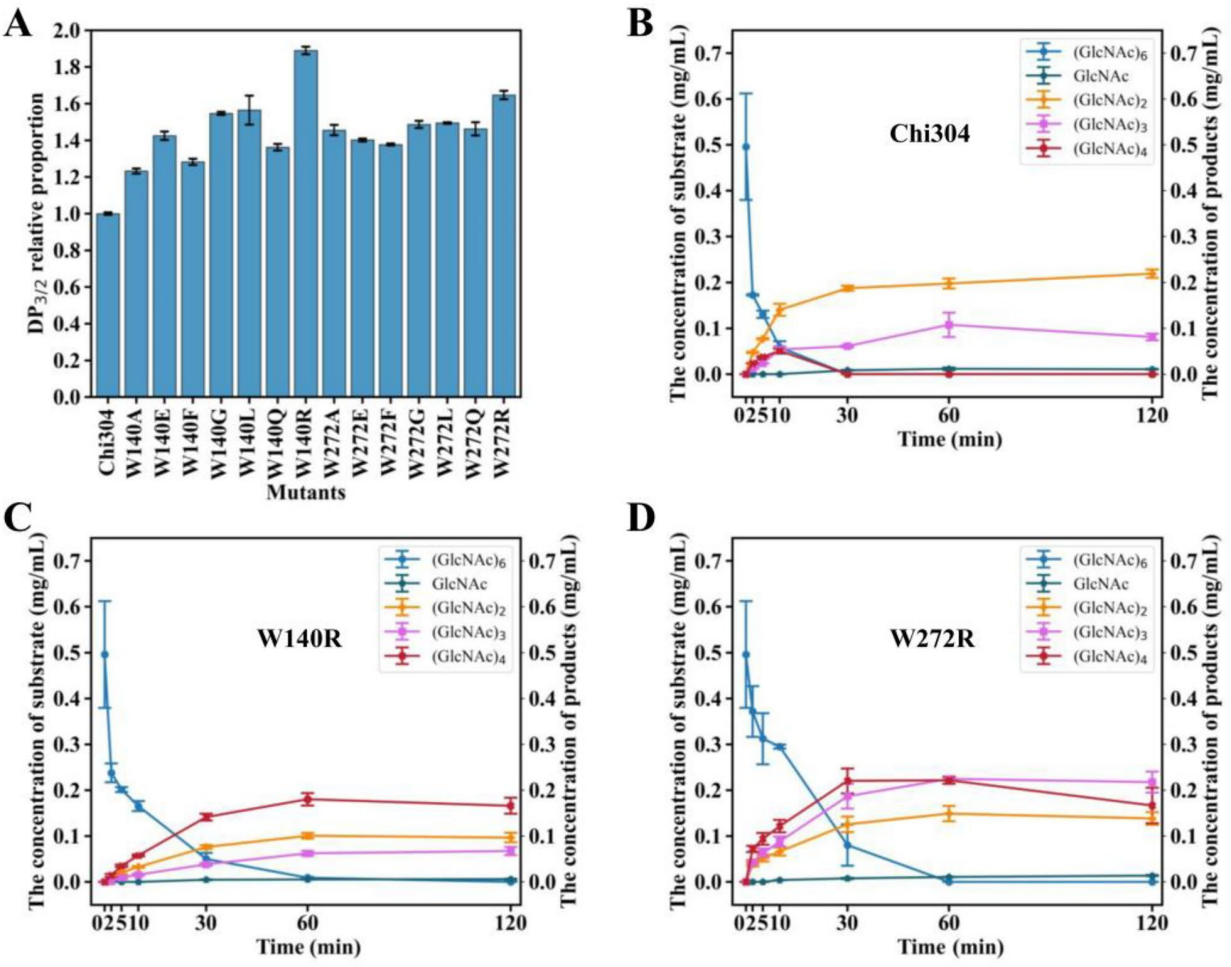
Validation of the endo-activities of single-point mutants designed through structural analysis. **A** The DP_3/2_ proportion of degradation products of colloidal chitin produced by Chi304 and its mutants; **B** time-course analysis of Chi304 degrading (GlcNAc)_6_; **C** time-course analysis of W140R degrading (GlcNAc)_6_; and **D** time-course analysis of W272R degrading (GlcNAc)_6_

The 14 single-point mutants noted above were constructed and successfully expressed in *E*. *coli* (Additional file 1: Fig. S1A). The enzymatic activity of each mutant was assessed using the dinitrosalicylic acid method at 85°C and pH 9.0 (Zhang et al. 2022). The activities of all mutants were reduced (Additional file 1: Fig. S1B), indicating the importance of both of the tryptophans to catalytic efficiency. To detect changes in cleavage mode and eliminate interference due to decreased activity, Chi304 and its mutants were diluted to 0.25 U for hydrolysis of colloidal chitin. All chitooligosaccharide products were detected through HPLC. The results showed that the degradation products were mainly *N*-acetylglucosamine, diacetyl-chitobiose and triacetyl-chitotriose. Thus, the ratio of triacetyl-chitotriose to diacetyl-chitobiose quantity (DP_3/2_) was used to further evaluate the changes in endo-activities of the mutants and the results showed that all mutants increased to varying degrees compared to the wild type. W140R and W272R showed the greatest increases, reaching DP_3/2_ values 1.89 and 1.65 times that of Chi304, respectively (Fig. 2A).

**Fig. 3:**
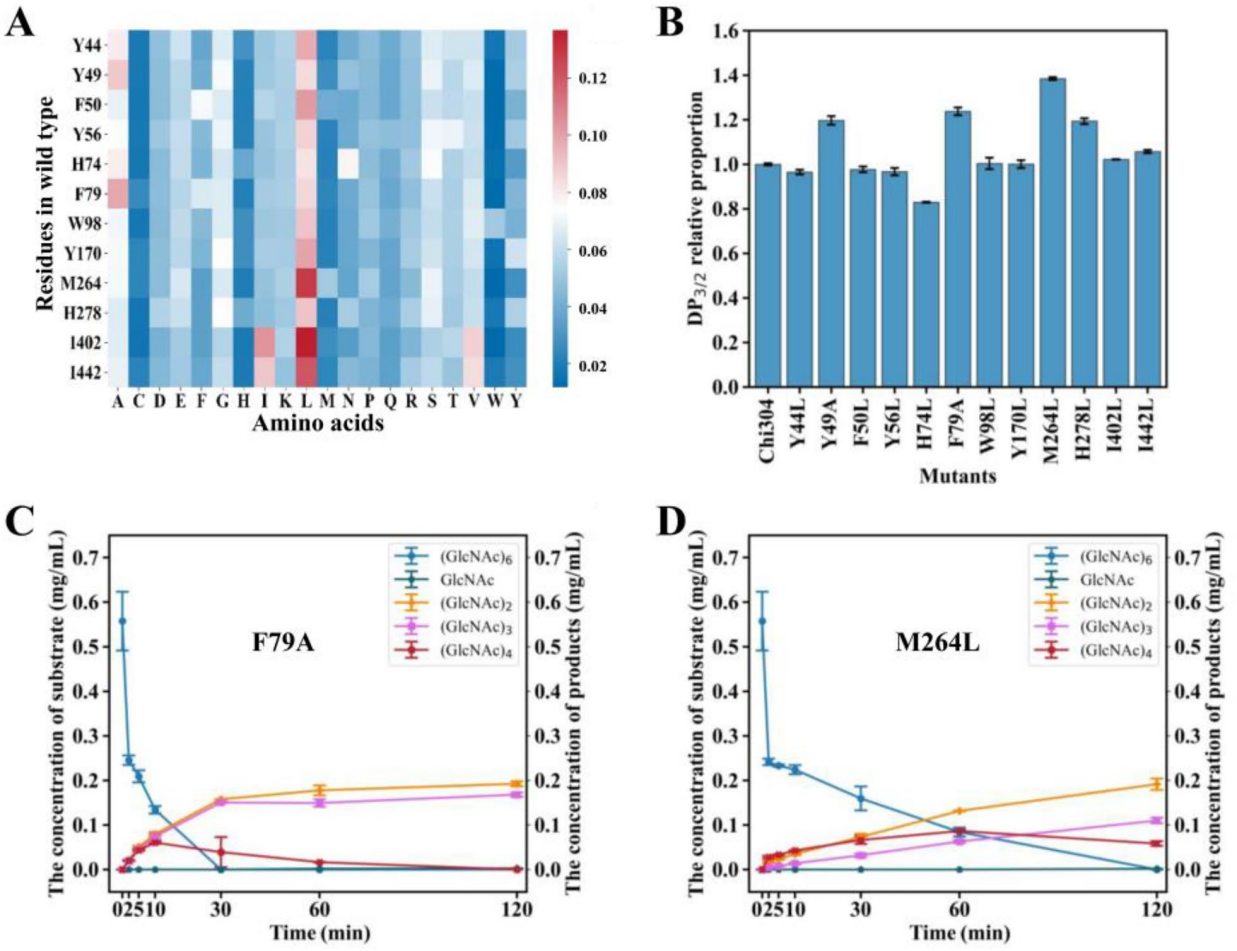
Validation of the endo-activities of single-point mutants identified through evolutionary analysis. **A** Analysis of amino acid frequency at selected mutation sites. **B** DP_3/2_ proportion of degradation products of colloidal chitin produced by Chi304 and its mutants; **C** time-course analysis of F79A degrading (GlcNAc)_6_; and **D** time-course analysis of M264L degrading (GlcNAc)_6_

The substrate (GlcNAc)_6_ was used to evaluate the cutting pattern of Chi304 and its mutants. Since there was nearly no N-acetylglucosamine produced in the whole process, the possible exo-cutting mode was DP2 + DP4 and the possible endo-cutting mode was DP3 + DP3 (Fig. 2B–D). As shown in Fig. 2B, for wild-type Chi304, exo-activity was predominant. As (GlcNAc)_6_ was gradually hydrolyzed, the amounts of (GlcNAc)_2_, (GlcNAc)_3_, and (GlcNAc)_4_ increased gradually. The product (GlcNAc)_2_ was predominant, suggesting strong exo-activity of Chi304. At 10 min, the amount of (GlcNAc)_4_ peaked, after which it began to be hydrolyzed and was consumed by 30 min. At 60 min, the amount of (GlcNAc)_3_ was greatest, following which a small amount was hydrolyzed (Fig. 2B). The amount of (GlcNAc)_4_ produced by W140R and W272R increased continually, with slight decreases after 60 min and 30 min, respectively, suggesting that the exo-activities of both W140R and W272R were attenuated (Figs. 2C and 3D). For W272R, the amount of (GlcNAc)_3_ continued to increase until 60 min, and then remained unchanged over the next hour. Furthermore, the yield of (GlcNAc)_3_ was 2.09 times that of Chi304 at 60 min (its peak), indicating enhancement of the DP3 + DP3 endo-cleavage mode (Figs. 2B and 3D). Thus, in the determination of the end product after 120 min, (GlcNAc)_2_ was the main product produced by Chi304. In the mutants W140R and W272R, (GlcNAc)_4_ accumulated abundantly due to the weakened exo-activity, while the accumulation of (GlcNAc)_3_ in the mutants of W272R demonstrated enhanced endo-activity (Additional file 1: Fig. S2).

### Evolutionary analysis with GH19-family chitinases for Chi304 modification

To further enhance the endo-cleavage activity of Chi304, evolutionary analysis aimed at identifying the disparity between Chi304 and chitinases derived from the glycoside hydrolase 19 family was conducted (Additional file 1: Table S2). The 14 most conserved amino acid sites were selected for mutation (Fig. 3A, Table 2, and Additional file 1: Table S2). After successful expression and purification in *E*. *coli* (Additional file 1: Fig. S3A), the enzymatic activity and composition of the products were detected as described above. The enzymatic activities of all mutants were reduced, with W53L and H269L showing the sharpest reductions, almost no enzymatic activity and discarded (Additional file 1: Fig. S3B). Among the degradation products of colloidal chitin, DP_3/2_ values of the products from the mutants F79A and M264L were 1.24 and 1.38 times that of Chi304, respectively (Fig. 3B).

**Table 2:** Mutants identified through evolutionary analysis

No.	Site	AA (WT)	Max. AA (GH19)	Diff. score
1	264	M	L	0.0768
2	278	H	L	0.0511
3	44	Y	L	0.0452
4	74	H	L	0.0446
5	98	W	L	0.0385
6	170	Y	L	0.0372
7	49	Y	A	0.0355
8	56	Y	L	0.0352
9	79	F	A	0.0335
10	269	H	L	0.0335
11	402	I	L	0.0333
12	53	W	L	0.0327
13	442	I	L	0.0309
14	50	F	L	0.0300

The top two mutants, F79A and M264L, were selected for analysis of the specific hydrolysis mechanism of (GlcNAc)_6_ over 120 min. (GlcNAc)_6_ was degraded thoroughly by F79A within 30 min. The main products, (GlcNAc)_2_ and (GlcNAc)_3_, increased continually. The amount of (GlcNAc)_3_ at 120 min was 2.08 times that of wild-type Chi304 (Fig. 3C and Additional file 1: Fig. S4), suggesting enhancement of the endo-cutting mode of DP3 + DP3. For M264L, the trend of product generation was similar to that of F79A, with (GlcNAc)_3_ production at 120 min that was 1.36 times that of wild-type Chi304 (Fig. 3D and Additional file 1: Fig. S4). In addition, (GlcNAc)_4_ accumulated among the degradation products of M264L (Additional file 1: Fig. S4), demonstrating an increase in endo-activity accompanied by weakened exo-activity.

### Combinatorial mutagenesis to alter the cleavage mode of Chi304

**Fig. 4:**
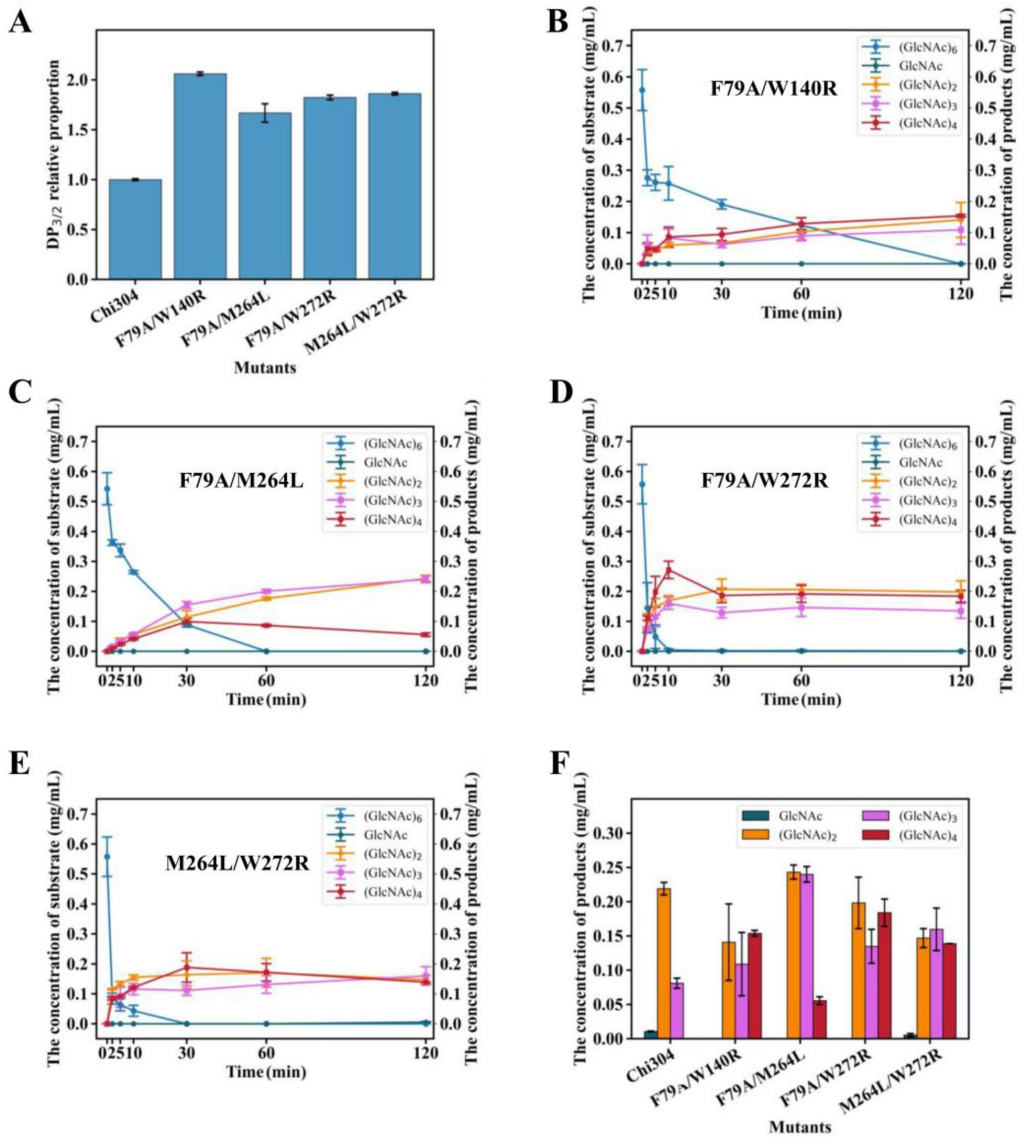
Validation of the endo-activities of double-point mutants. **A** DP_3/2_ proportion of degradation products of colloidal chitin produced by Chi304 and its double-point mutants; **B** time-course analysis of F79A/W140R degrading (GlcNAc)_6_; **C** time-course analysis of F79A/M264L degrading (GlcNAc)_6_; **D** time-course analysis of F79A/M272R degrading (GlcNAc)_6_; **E** time-course analysis of M264L/W272R degrading (GlcNAc)_6_; **F** product composition analysis of (GlcNAc)_6_ degradation by Chi304 and its double-point mutants at 120 min

To further improve the endo-activity of Chi304, the apparently positive single-point mutants obtained above, namely F79A, M264L, W140R, and W272R, were combined randomly to obtain double-point mutants, i.e., F79A/M264L, F79A/W140R, F79A/W272R, W140R/M264L, M264L/W272R, and W140R/W272R. After expression and purification (Additional file 1: Fig. S5A), the remaining activities of these mutants were measured. The activities of all mutants decreased dramatically, especially for W140R/M264L and W140R/W272R, which retained only 5.7% and 2.6% of the wild-type activity, respectively (Additional file 1: Fig. S5B). Therefore, these two mutants were discarded for subsequent validation. When degrading colloidal chitin, the DP_3/2_ ratios of degradation products of the four double-point mutants (F79A/W140R, F79A/M264L, F79A/W272R, and M264L/W272R) were 2.06, 1.67, 1.82, and 1.86 times of that Chi304, respectively, indicating that all double-point mutants had higher endo-activities than the wild type (Fig. 4A).

To further compare the endo-activities of the four double-point mutants, hydrolysis of (GlcNAc)_6_ was also performed for 120 min. As shown in Fig. 4B, F79A/W140R hydrolyzed (GlcNAc)_6_ at a reduced rate and had fully consumed the substrate at 120 min. The yields of (GlcNAc)_2–4_ were generally consistent and low (Fig. 4B). For the mutant F79A/M264L, the degradation product (GlcNAc)_3_ increased throughout measurement. (GlcNAc)_2_ showed the same trend, while the production of (GlcNAc)_4_ first increased and then decreased, with a peak at 30 min (Fig. 4C). By contrast, F79A/W272R and M264L/W272R showed higher (GlcNAc)_6_ hydrolysis activities, completely degrading this substrate within 10 min and 30 min, respectively. Levels of the products (GlcNAc)_2–3_ continually increased, with (GlcNAc)_4_ showing slight decreases at 10 min and 30 min, respectively (Fig. 4D and E). From the composition of products at 120 min produced by the four mutants, compared to Chi304, significant increases in (GlcNAc)_3_, (GlcNAc)_4_, and especially (GlcNAc)_4_, were observed, indicating that the exo-activity of all four mutants was reduced (Fig. 4F). The quantity of (GlcNAc)_3_ produced was 1.35, 2.96, 1.67, and 1.97 times that of Chi304, respectively (Fig. 4F). The accumulation of (GlcNAc)_3_ demonstrated the enhanced endo-activities of the four double-point mutants in terms of (GlcNAc)_6_ hydrolysis.

### Production of highly polymerized functional chitooligosaccharides by the mutants

**Fig. 5:**
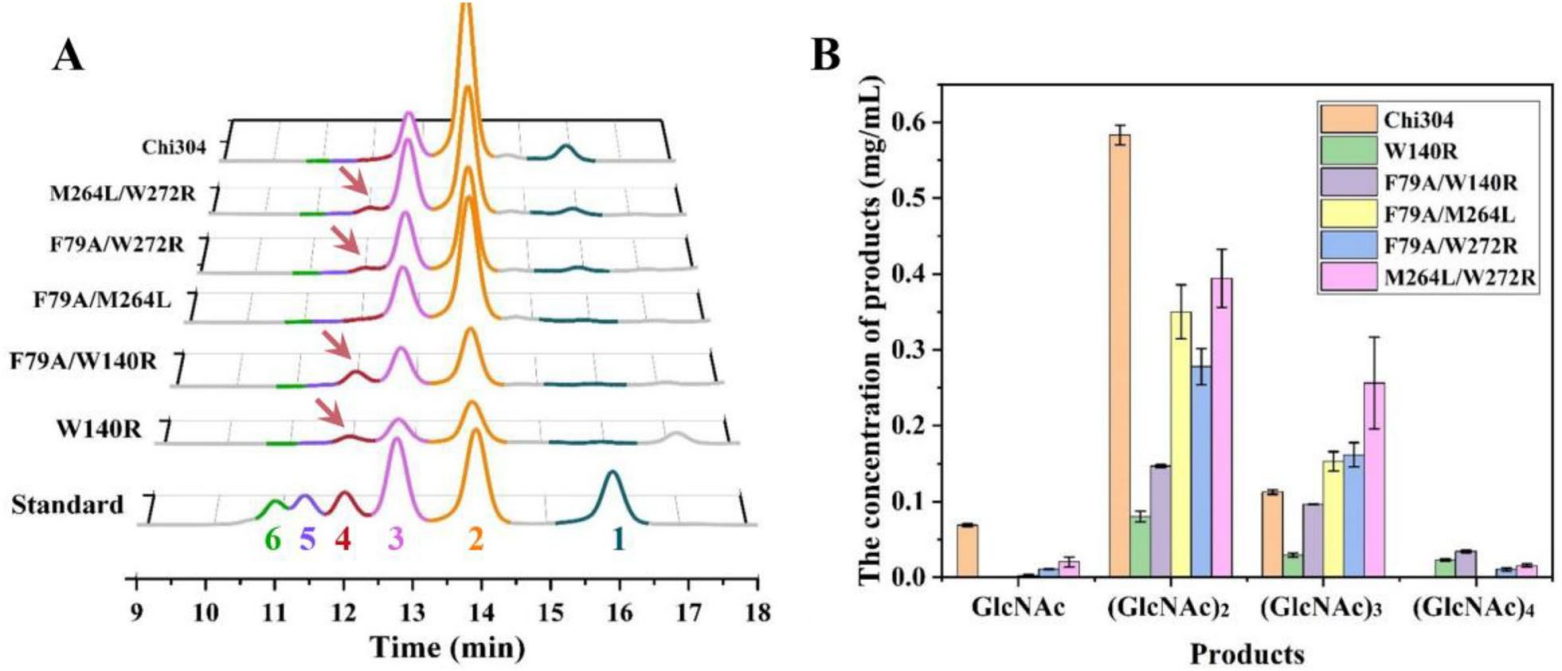
Analysis of the degradation products of colloidal chitin produced by superior mutants. **A** HPLC analysis of the components of the degradation products; and **B** quantitative analysis of the components of the products

To produce functional chitooligosaccharides with DP ≥ 3, we selected the superior mutant W140R and the four double-point mutants described above for degradation of colloidal chitin and assayed the composition of the products. The HPLC chromatogram indicated significant accumulation of (GlcNAc)_4_ in the experiments with W140R, F79A/W140R, F79A/W272R, and M264L/W272R, and this product was not detected from Chi304 or F79A/M264L (Fig. 5A). Further quantitative analysis showed that F79A/W140R accumulated the most (GlcNAc)_4_, reaching levels 1.51, 3.22, and 2.21 times those of W140R, F79A/W272R, and M264L/W272R, respectively (Fig. 5B). Meanwhile, M264L/W272R was the best producer of (GlcNAc)_3_, with a yield 2.28 times that of the wild type (Fig. 5B). For the two mutants that produced highly polymerized chitooligosaccharides, F79A/W140R and M264L/W272R, the contents of both GlcNAc and (GlcNAc)_2_ were dramatically reduced compared with Chi304 (Fig. 5B). In addition, we determined the *T*_m_ values and optimal reaction temperatures of W140R, F79A/W140R, and M264L/W272R. The results showed that the *T*_m_ values were 90.85 ± 0.16 ℃, 90.65 ± 0.14 ℃ and 86.74 ± 0.19 ℃, respectively (Additional file 1: Fig. S6A). The optimum reaction temperatures for W140R, F79A/W140R and M264L/W272R to degrade colloidal chitin were 80–85 ℃ (Additional file 1: Fig. S6B), which were similar to that of the wild type (Zhang et al. 2022). Therefore, the endo-active mutants retained the thermophilic properties.

### Structural investigation of variants exhibiting increased endo-activity

**Fig. 6:**
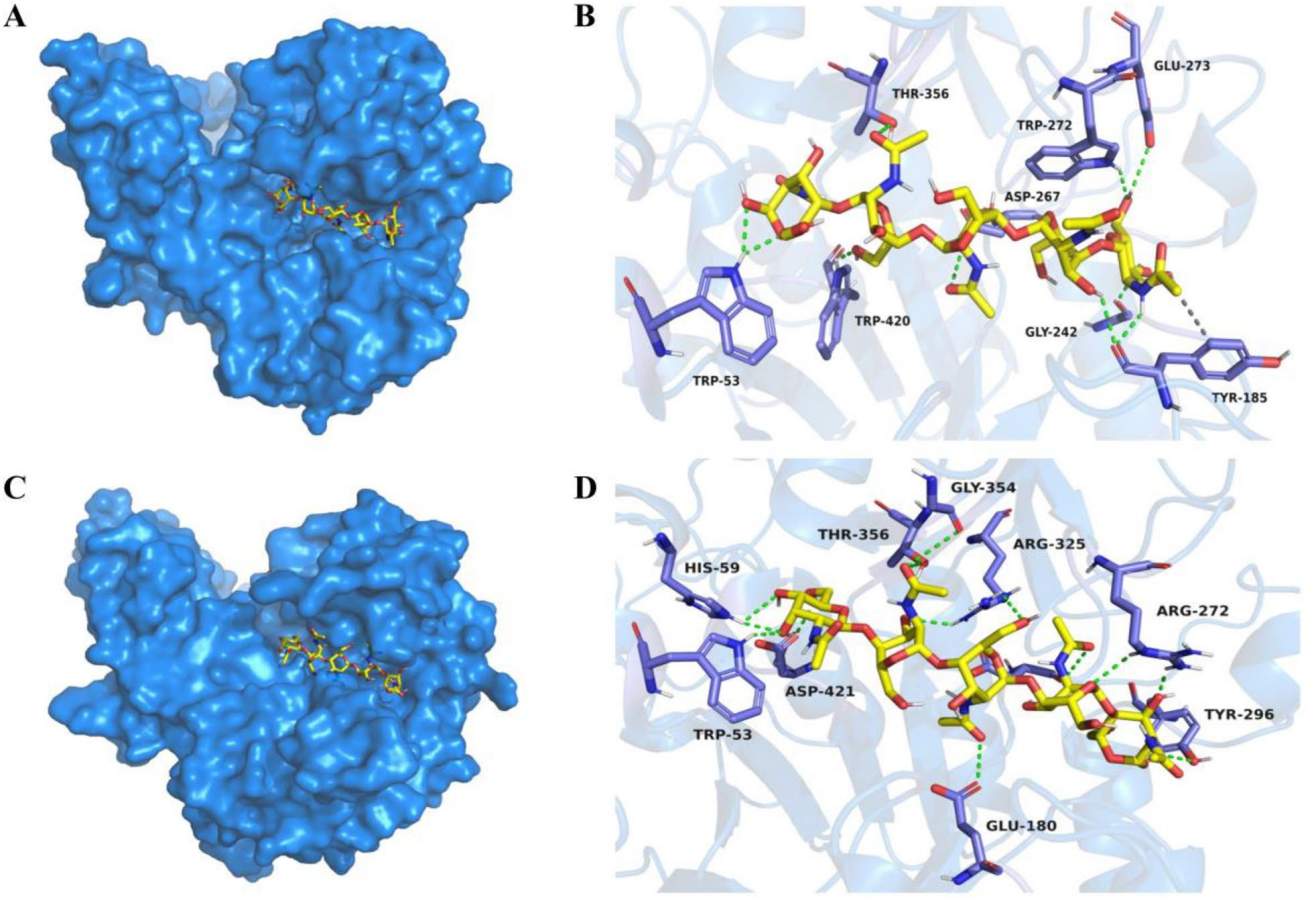
Docking and interaction analysis between the homologous structures of the double-point mutants and penta-*N*-acetylchitopentaose. **A** Homologous structure of F79A/W140R docked with penta-*N*-acetylchitopentaose; **B** analysis of the interaction between F79A/W140R and the substrate; **C** homologous structure of M264L/W272R docked with penta-*N*-acetylchitopentaose; **D** analysis of the interaction between M264L/W272R and the substrate. Dotted green line: hydrogen bond; dotted grey line: hydrophobic interaction

To analyze the mechanism through which cleavage modes of F79A/W140R and M264L/W272R were altered, we first obtained their structures through homology modeling. Molecular docking analyses were then performed using penta-N-acetylchitopentaose as the substrate. As illustrated in Fig. 6A and C, the two mutants exhibited more exposed substrate-binding pockets than that of Chi304, which might affect substrate entry and exit (Li et al. 2022). Besides, we found that the size of the binding pockets predicted using the P2Rank (https://prankweb.cz/) (Jakubec et al. 2022) and calculated by Schrödinger's App (Fowler and Morain 2020) was decreased from 355.35 Å^3^ (Chi304) to 283.66 Å^3^ (F79A/W140R) and 242.84 Å^3^ (M264L/W272R), respectively. Endo-cleavage occurs more frequently when mutants have shallower and smaller pockets (Baban et al. 2010). Furthermore, there were six hydrogen bonds in the wild type (Additional file 1: Fig. S7). While in the F79A/W140R mutant, a total of ten hydrogen bonds and one hydrophobic interaction were formed between the substrate and surrounding amino acids within 3.5 Å (Fig. 6B). Besides, there were 13 hydrogen bonds formed within the same distance range in the M264L/W272R mutant (Fig. 6D). Consequently, both mutants had a higher interaction force than the wild type, which could aid the chitinase in binding the substrate to the shallower pockets, thus enhancing the endo-cutting activity (Jiménez-Ortega et al. 2022).

## Discussion

In this study, we aimed to get an excellent chitinase endowed with endo-activity and thermostability, which was of great importance to produce highly polymerized chitooligosaccharides in industry eco-friendlily. The thermophilic chitinase, Chi304, exhibited excellent thermal stability at 80 and 90 °C and the optimum temperature towards chitin was up to be 85 ℃ (Zhang et al. 2022). Besides, Chi304 had some endo-activity and therefore it was a good chassis to be modified to enhance endo-activity.

According to our process, evolutionary analysis was conducted to predict the conserved amino acids related to endo-activities of chitinases. OsChia1c, which is an endo-chitinase (GH19 family) derived from rice, *Oryza sativa* L, was used as a template to build a data set. Then, we calculated the position-specific amino acid probability (PSAP) to search for the conserved amino acids in these sequences. The results showed that 12 out of 14 mutants tent to be leucine (L), although two of them (M264L and H278L) and the other two alanine mutants (Y49A and F79A) endowed with increased endo-activity. According to the previous study, leucine rich might be helpful in protein–protein or protein–substrate interactions and the structure could be used as a model to study the interactions of other leucine-rich proteins with their ligands (Dixon et al. 2014; Kobe and Deisenhofer 1995). In addition, Y49 and F79, located within the substrate-binding pocket, were mutated into alanine (A) with a smaller side chain, potentially increasing the substrate-binding pocket. Therefore, the strategy used in our process was reasonable and effective, but since these mutated sites severely affected the catalytic efficiency of Chi304, the enhancement of endo-activity was not extremely significant. In the future, we will continue to focus on chitinases or mutants with both improved endo-activity and catalytic efficiency to produce chitooligosaccharides with a high degree of polymerization (DP ≥ 3).

## Conclusion

In this study, two strategies for enhancing the endo-activity of Chi304 were employed using structural analysis and evolutionary analysis. Structural analysis showed that W140 and W272 helped to form a deep binding cleft. Mutation of these residues to R reduced the exo-activity and increased the endo-activity of W272R. Through evolutionary analysis of GH19-family chitinases, F79A and M264L were identified as mutations enhancing endo-activity. These four favorable mutation sites were combined randomly to construct double-point mutants. Degradation of colloidal chitin and (GlcNAc)_6_ demonstrated that all four double-point mutants (F79A/W140R, F79A/M264L, F79A/W272R, and M264L/W272R) were endowed with enhanced endo-activity and weakened exo-activity. When colloidal chitin was used as a substrate, F79A/W140R accumulated the most (GlcNAc)_4_, and M264L/W272R was the best producer of (GlcNAc)_3_, with (GlcNAc)_3_ yield 2.28 times that of the wild type. Overall, these synergistic changes were conducive to producing chitooligosaccharides (DP ≥ 3), and the theoretical concept developed here may be used to modify the substrate cleavage patterns of other glucoside hydrolases.

### Supplementary Information

**Additional file 1: Fig. S1.** The detection of expression and enzymatic activity of the single-point mutants from structure analysis. (a) The SDS-PAGE of purified protein Chi304 and mutants. M: maker, 1: Chi304, 2: W140A, 3: W140E, 4: W140F, 5: W140G, 6: W140L, 7: W140Q, 8: W140R, 9: W272A, 10: W272E, 11: W272F, 12: W272G, 13: W272L, 14: W272Q, 15: W272R. (b) Relative enzymatic activity of Chi304 and mutants. **Fig. S2.** Product composition analysis of (GlcNAc)_6_ degradation by Chi304, W140R and W272R at 120 min. **Fig. S3.** The detection of expression and enzymatic activity of the single-point mutants from evolutionary analysis. (a) The SDS-PAGE of purified protein Chi304 and mutants. M: maker, 1: Chi304, 2: Y44L, 3: Y49A, 4: F50L, 5: Y56L, 6: H74L, 7: F79A, 8: W98L, 9: Y170L, 10: M264L, 11: H278L, 12: I402L, 13: I442L. (b) Relative enzymatic activity of Chi304 and mutants. **Fig. S4.** Product composition analysis of (GlcNAc)_6_ degradation by Chi304, F79A and M264L at 120 min. **Fig. S5.** The detection of expression and enzymatic activity detection of the double - point mutants. (a) The SDS-PAGE of purified protein Chi304 and mutants. M: maker, 1: Chi304, 2: F79A/W140A, 3: F79A/M264L, 4: F79A/W272R, 5: W140R/M264L, 6: W140R/W272R, 7: M264L/W272R. (b) Relative enzymatic activity of double point mutants. **Fig. S6.** The detection of *T*_m_ values (A) and optimum reaction temperatures (B) of mutants. **Fig. S7.** The analysis of the hydrogen bond between Chi304 and the substrate. **Table S1.** The primers used in this study. **Table S2.** The value of the Position-Specific Amino-acid Probability (PSAP) of Chi304 alignment to chitinases in the GH19 family.

## Data Availability

Data will be made available on request.
