# Supplementary material for: Enhancing the endo-activity of the thermophilic chitinase to yield chitooligosaccharides with high degrees of polymerization
Source: Bioresour Bioprocess. 2024 Mar 7;11(1):29. doi: 10.1186/s40643-024-00735-x (PMC10991111; doi:10.1186/s40643-024-00735-x)
Supplement: Supplementary file 1 — Additional file 1: Fig. S1. The detection of expression and enzymatic activity of the single-point mutants from structure analysis. (a) The SDS-PAGE of purified protein Chi304 and mutants. M: maker, 1: Chi304, 2: W140A, 3: W140E, 4: W140F, 5: W140G, 6: W140L, 7: W140Q, 8: W140R, 9: W272A, 10: W272E, 11: W272F, 12: W272G, 13: W272L, 14: W272Q, 15: W272R. (b) Relative enzymatic activity of Chi304 and mutants. Fig. S2. Product composition analysis of (GlcNAc)6 degradation by Chi304, W140R and W272R at 120 min. Fig. S3. The detection of expression and enzymatic activity of the single-point mutants from evolutionary analysis. (a) The SDS-PAGE of purified protein Chi304 and mutants. M: maker, 1: Chi304, 2: Y44L, 3: Y49A, 4: F50L, 5: Y56L, 6: H74L, 7: F79A, 8: W98L, 9: Y170L, 10: M264L, 11: H278L, 12: I402L, 13: I442L. (b) Relative enzymatic activity of Chi304 and mutants. Fig. S4. Product composition analysis of (GlcNAc)6 degradation by Chi304, F79A and M264L at 120 min. Fig. S5. The detection of expression and enzymatic activity detection of the double - point mutants. (a) The SDS-PAGE of purified protein Chi304 and mutants. M: maker, 1: Chi304, 2: F79A/W140A, 3: F79A/M264L, 4: F79A/W272R, 5: W140R/M264L, 6: W140R/W272R, 7: M264L/W272R. (b) Relative enzymatic activity of double point mutants. Fig. S6. The detection of Tm values (A) and optimum reaction temperatures (B) of mutants. Fig. S7. The analysis of the hydrogen bond between Chi304 and the substrate. Table S1. The primers used in this study. Table S2. The value of the Position-Specific Amino-acid Probability (PSAP) of Chi304 alignment to chitinases in the GH19 family. [file 40643_2024_735_MOESM1_ESM.doc]

Additional Materials for

**Enhancing the endo-activity of the thermophilic chitinase to yield chitooligosaccharides with high degrees of polymerization**

# Feifei Guan *et al*.

*Corresponding author. Email: [huanghuoqing@caas.cn](mailto:huanghuoqing@caas.cn)

**This PDF file includes:**

Figs. S1 to S7

Tables S1 and S2


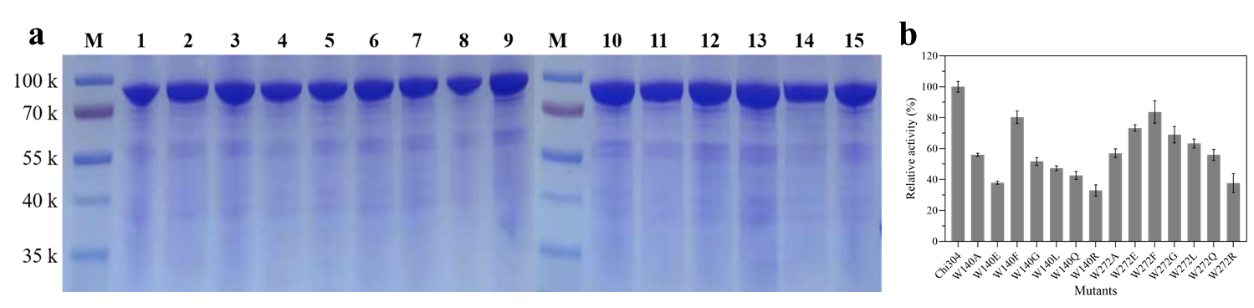


Fig. S1 The detection of expression and enzymatic activity of the single-point mutants from structure analysis

(a) The SDS-PAGE of purified protein Chi304 and mutants. M: maker, 1: Chi304, 2: W140A, 3: W140E, 4: W140F, 5: W140G, 6: W140L, 7: W140Q, 8: W140R, 9: W272A, 10: W272E, 11: W272F, 12: W272G, 13: W272L, 14: W272Q, 15: W272R. (b) Relative enzymatic activity of Chi304 and mutants.


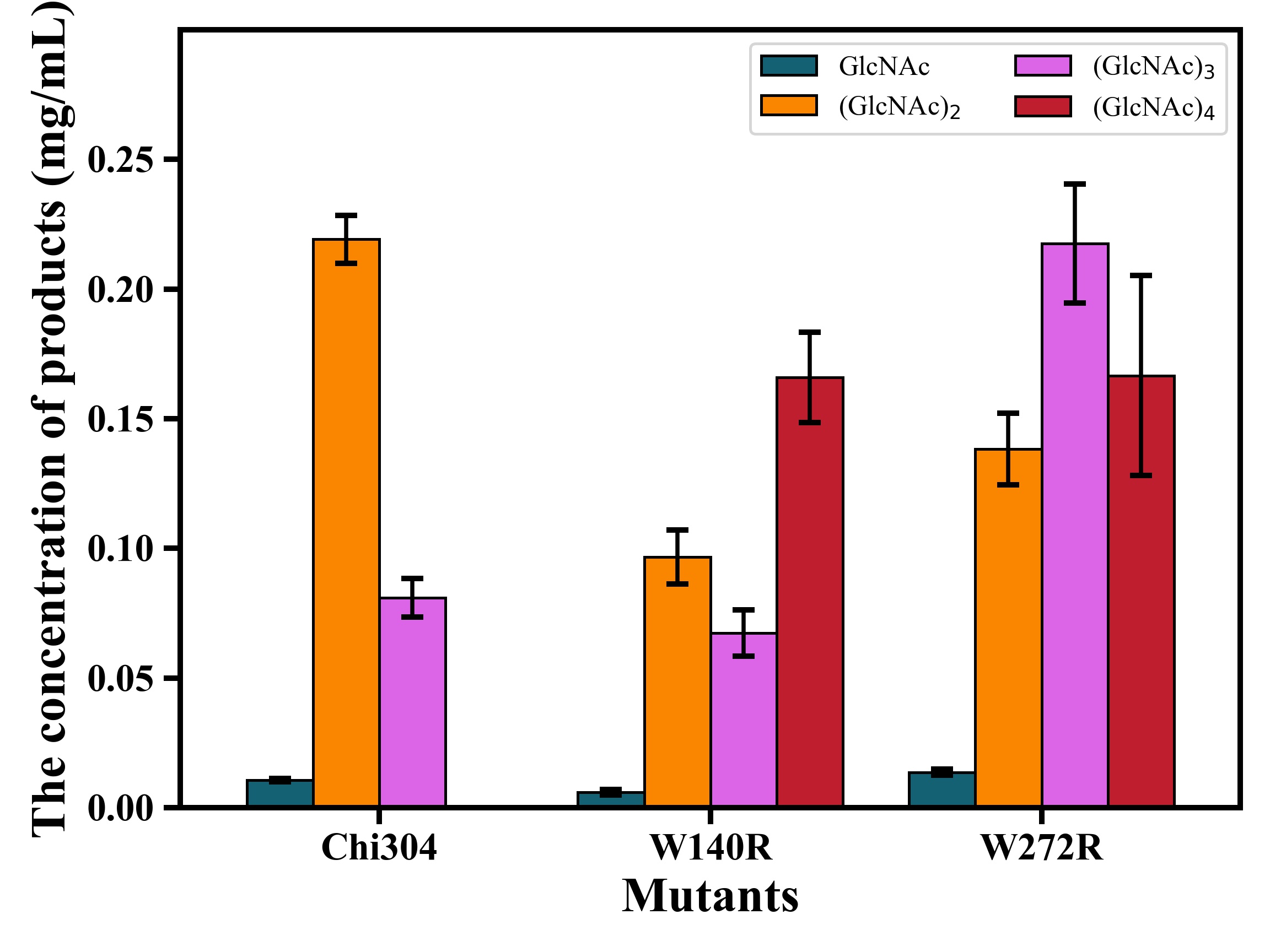


Fig. S2 Product composition analysis of (GlcNAc)_6_ degradation by Chi304, W140R and W272R at 120 min.


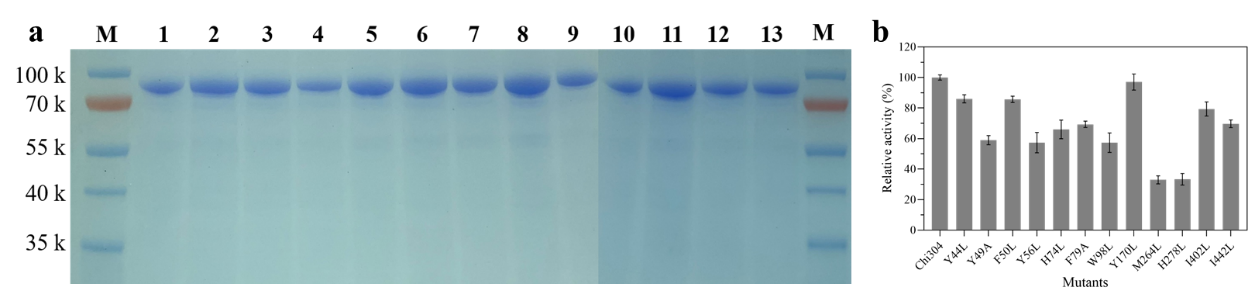


Fig. S3 The detection of expression and enzymatic activity of the single-point mutants from evolutionary analysis

(a) The SDS-PAGE of purified protein Chi304 and mutants. M: maker, 1: Chi304, 2: Y44L, 3: Y49A, 4: F50L, 5: Y56L, 6: H74L, 7: F79A, 8: W98L, 9: Y170L, 10: M264L, 11: H278L, 12: I402L, 13: I442L. (b) Relative enzymatic activity of Chi304 and mutants.


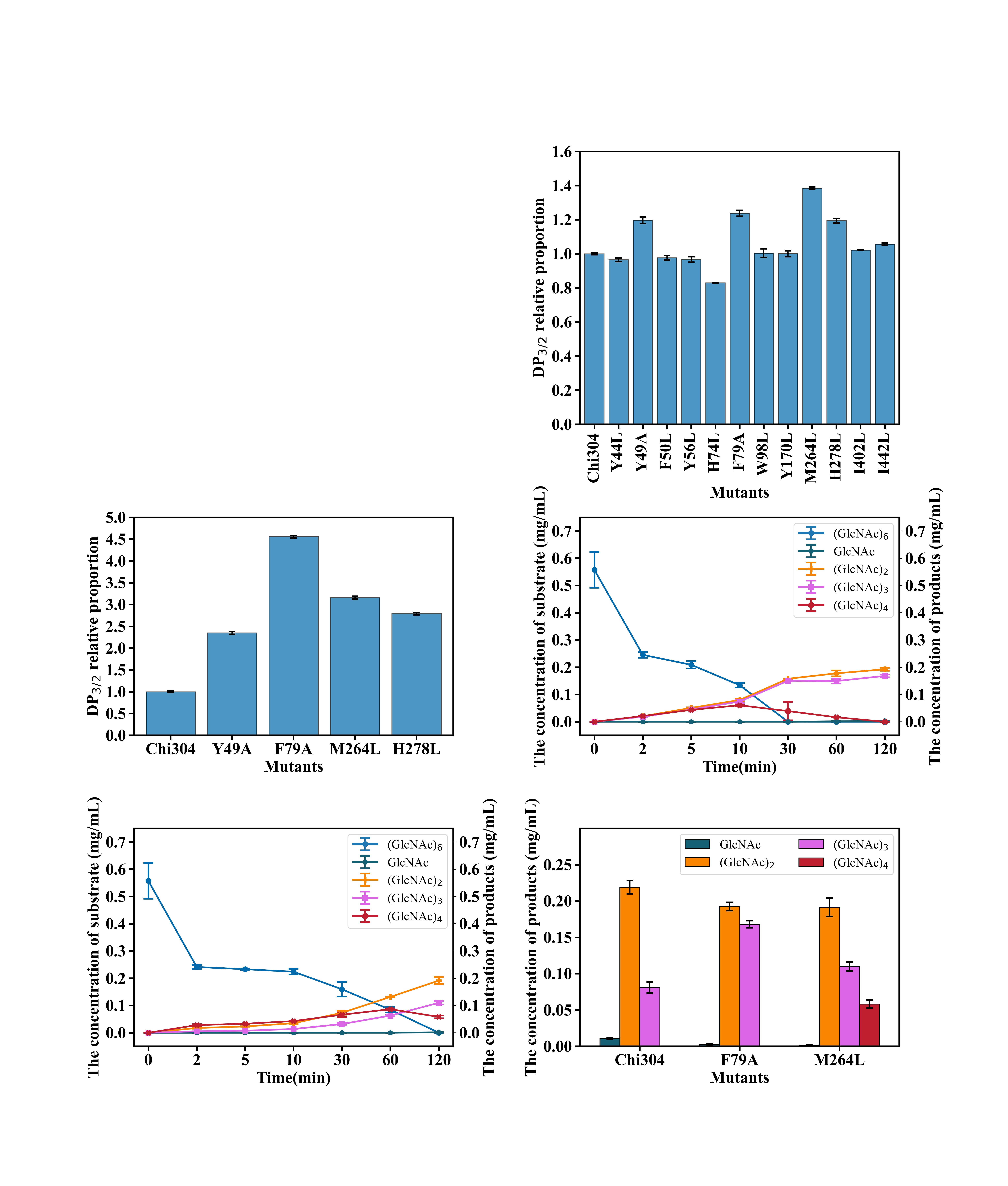


Fig. S4 Product composition analysis of (GlcNAc)_6_ degradation by Chi304, F79A and M264L at 120 min.


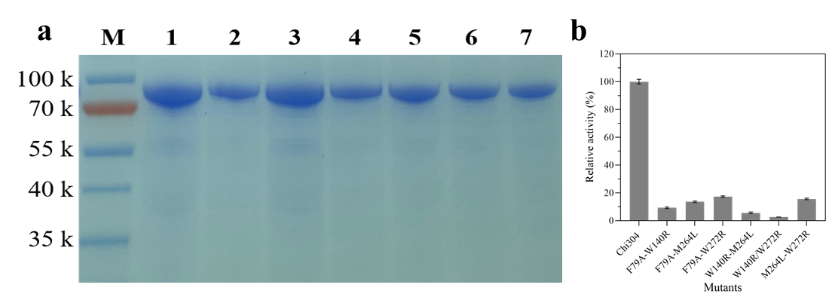


Fig. S5 The detection of expression and enzymatic activity detection of the double - point mutants

(a) The SDS-PAGE of purified protein Chi304 and mutants. M: maker, 1: Chi304, 2: F79A/W140A, 3: F79A/M264L, 4: F79A/W272R, 5: W140R/M264L, 6: W140R/W272R, 7: M264L/W272R. (b) Relative enzymatic activity of double point mutants.


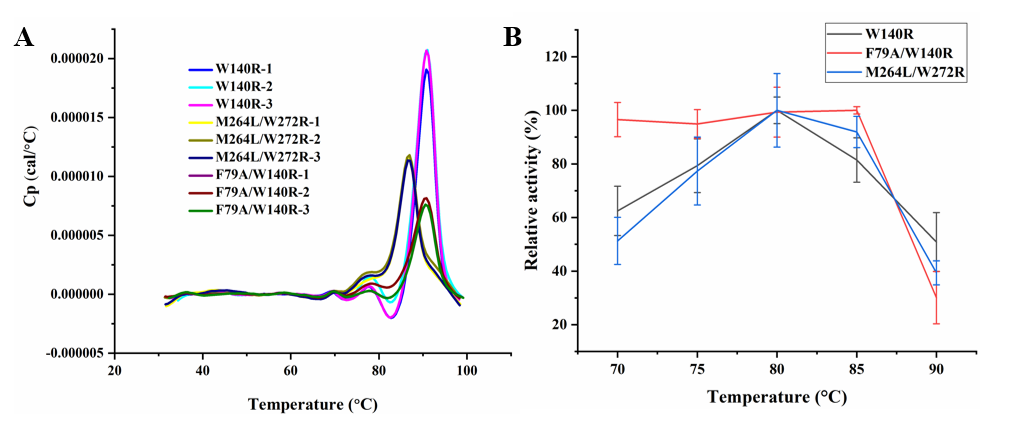


Figure S6 The detection of *T*_m_ values (A) and optimum reaction temperatures (B) of mutants


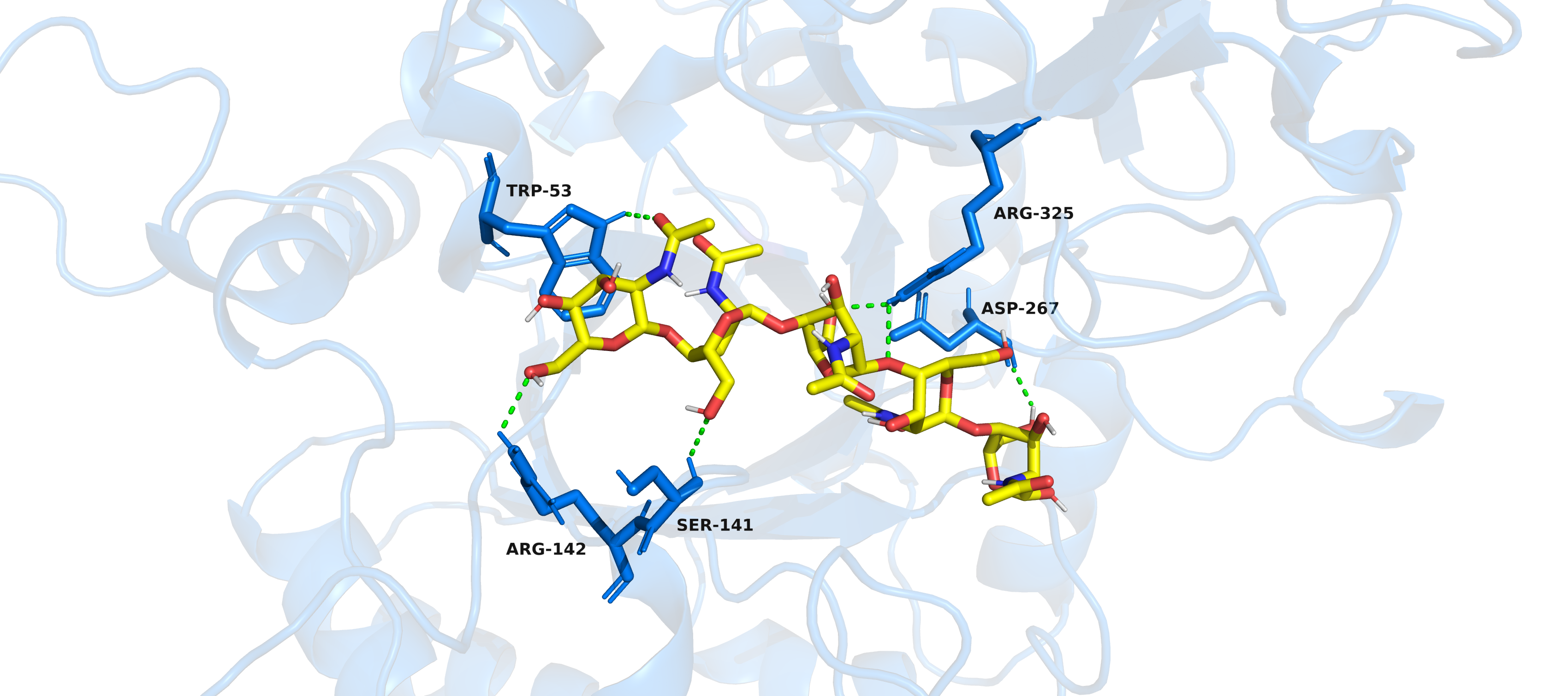


Fig. S7 The analysis of the hydrogen bond between Chi304 and the substrate.

Table S1 The primers used in this study

| Primers | Sequence (5’—3’) |
| --- | --- |
| T7 | TAATACGACTCACTATAGGG |
| T7ter | GCTAGTTATTGCTCAGCGG |
| JC360-F | CGTATTACCAAACCCGTGCG |
| JC360-R | CGCACGGGTTTGGTAATACG |
| Y44L-F(Chi304) | CGGGTGGCCTGCCG(TAC)ctgCACATCGTGGTTTATTTC |
| Y49A-R(Chi304) | GTAAATGCCCCATTCCGGGAA(ATA)cgcAACCACGATGTGG |
| F50L-F(Chi304) | CACATCGTGGTTTAT(TTC)ctgCCGGAATGGGGCATTTACG |
| W53L-F(Chi304) | GGTTTATTTCCCGGAA(TGG)ctgGGCATTTACGCGGGTCAC |
| Y56L-F(Chi304) | CCCGGAATGGGGCATT(TAC)ctgGCGGGTCACAACTAC |
| H74L-F(Chi304) | GTTCGAATACATCACC(CAC)ctgCTGAACTATGCGTTTC |
| F79A-R(Chi304) | CACTTTCTTAATCTCCAG(AAA)cgcCGCATAGTTCAGGTGG |
| W98L-F(Chi304) | GGTATCATTGACAGC(TGG)ctgGCGAGCCTGCAGAAAG |
| Y170L-R(Chi304) | GTCGATGCCATCGTAACC(ATA)cagGGTACGCAGGTAC |
| M264L-R(Chi304) | CCGTGGAAATCGTAGGT(CAT)cagCAGACCAATATAGTC |
| H269L-R(Chi304) | GGTAACGTTCTCCCAGCCAC(GTG)cagGGAAATCGTAGGTC |
| H278L-R(Chi304) | GTTCGGATACAGCGGGCTTTG(CAC)cagGTTGGTAACGTTC |
| I402L-F(Chi304) | CTTCGAGGATCTGGAAAGC(ATC)ctgACCATTAAGGCGAAC |
| I442L-F(Chi304) | GCGGAACTGACCAAAGCG(ATC)ctgTATGAACTGTTTGGTG |
| W140A-R(Chi304) | GTAGCCGCTACGGCT(CCA)cgcGCCACCAACGCTGAAC |
| W140E-R(Chi304) | GTAGCCGCTACGGCT(CCA)ttcGCCACCAACGCTGAAC |
| W140F-R(Chi304) | GTAGCCGCTACGGCT(CCA)aaaGCCACCAACGCTGAAC |
| W140G-R(Chi304) | GTAGCCGCTACGGCT(CCA)gccGCCACCAACGCTGAAC |
| W140L-R(Chi304) | GTAGCCGCTACGGCT(CCA)cagGCCACCAACGCTGAAC |
| W140Q-R(Chi304) | GTAGCCGCTACGGCT(CCA)ctgGCCACCAACGCTGAAC |
| W140R-R(Chi304) | GTAGCCGCTACGGCT(CCA)gcgGCCACCAACGCTGAAC |
| W272A-R(Chi304) | CCGTGGAAATCGTAGGT(CAT)cgcCAGACCAATATAGTC |
| W272E-R(Chi304) | CCGTGGAAATCGTAGGT(CAT)ttcCAGACCAATATAGTC |
| W272F-R(Chi304) | CCGTGGAAATCGTAGGT(CAT)aaaCAGACCAATATAGTC |
| W272G-R(Chi304) | CCGTGGAAATCGTAGGT(CAT)gccCAGACCAATATAGTC |
| W272L-R(Chi304) | CCGTGGAAATCGTAGGT(CAT)cagCAGACCAATATAGTC |
| W272Q-R(Chi304) | CCGTGGAAATCGTAGGT(CAT)ctgCAGACCAATATAGTC |
| W272R-R(Chi304) | CCGTGGAAATCGTAGGT(CAT)gcgCAGACCAATATAGTC |

Table S2 The value of the Position-Specific Amino-acid Probability (PSAP) of Chi304 alignment to chitinases in the GH19 family

| **No** | **264** | **278** | **44** | **74** | **98** | **170** | **49** | **56** | **79** | **269** | **402** | **53** | **442** | **50** |
| --- | --- | --- | --- | --- | --- | --- | --- | --- | --- | --- | --- | --- | --- | --- |
| **AA** | M | H | Y | H | W | Y | Y | Y | F | H | I | W | I | F |
| **A** | 0.07 | 0.07 | 0.08 | 0.08 | 0.07 | 0.07 | 0.09 | 0.07 | 0.1 | 0.07 | 0.07 | 0.07 | 0.07 | 0.07 |
| **C** | 0.02 | 0.02 | 0.02 | 0.02 | 0.02 | 0.02 | 0.02 | 0.02 | 0.03 | 0.02 | 0.02 | 0.02 | 0.02 | 0.02 |
| **D** | 0.05 | 0.06 | 0.05 | 0.05 | 0.05 | 0.05 | 0.05 | 0.05 | 0.05 | 0.05 | 0.04 | 0.05 | 0.04 | 0.05 |
| **E** | 0.06 | 0.06 | 0.06 | 0.06 | 0.06 | 0.06 | 0.06 | 0.06 | 0.06 | 0.07 | 0.05 | 0.06 | 0.05 | 0.06 |
| **F** | 0.03 | 0.03 | 0.04 | 0.04 | 0.05 | 0.04 | 0.04 | 0.04 | 0.07 | 0.03 | 0.03 | 0.06 | 0.04 | 0.07 |
| **G** | 0.07 | 0.07 | 0.07 | 0.06 | 0.07 | 0.07 | 0.07 | 0.07 | 0.07 | 0.06 | 0.06 | 0.07 | 0.06 | 0.07 |
| **H** | 0.02 | 0.04 | 0.03 | 0.04 | 0.03 | 0.03 | 0.02 | 0.02 | 0.02 | 0.05 | 0.02 | 0.02 | 0.02 | 0.02 |
| **I** | 0.05 | 0.05 | 0.05 | 0.05 | 0.05 | 0.05 | 0.05 | 0.05 | 0.05 | 0.05 | 0.1 | 0.05 | 0.09 | 0.06 |
| **K** | 0.05 | 0.05 | 0.06 | 0.05 | 0.05 | 0.05 | 0.05 | 0.05 | 0.05 | 0.05 | 0.06 | 0.05 | 0.05 | 0.05 |
| **L** | 0.13 | 0.09 | 0.1 | 0.08 | 0.09 | 0.1 | 0.08 | 0.09 | 0.09 | 0.08 | 0.14 | 0.08 | 0.12 | 0.1 |
| **M** | 0.05 | 0.02 | 0.02 | 0.02 | 0.02 | 0.02 | 0.03 | 0.02 | 0.02 | 0.02 | 0.03 | 0.02 | 0.02 | 0.04 |
| **N** | 0.04 | 0.05 | 0.04 | 0.08 | 0.04 | 0.04 | 0.05 | 0.04 | 0.04 | 0.06 | 0.03 | 0.04 | 0.05 | 0.04 |
| **P** | 0.05 | 0.04 | 0.05 | 0.05 | 0.05 | 0.04 | 0.05 | 0.05 | 0.05 | 0.06 | 0.04 | 0.05 | 0.04 | 0.04 |
| **Q** | 0.04 | 0.04 | 0.04 | 0.04 | 0.04 | 0.04 | 0.04 | 0.05 | 0.04 | 0.04 | 0.03 | 0.04 | 0.04 | 0.04 |
| **R** | 0.05 | 0.05 | 0.05 | 0.05 | 0.05 | 0.05 | 0.05 | 0.05 | 0.05 | 0.05 | 0.04 | 0.05 | 0.05 | 0.05 |
| **S** | 0.07 | 0.07 | 0.07 | 0.07 | 0.06 | 0.06 | 0.07 | 0.07 | 0.06 | 0.07 | 0.05 | 0.06 | 0.06 | 0.06 |
| **T** | 0.05 | 0.05 | 0.06 | 0.06 | 0.05 | 0.05 | 0.06 | 0.07 | 0.05 | 0.07 | 0.06 | 0.06 | 0.05 | 0.06 |
| **V** | 0.06 | 0.06 | 0.06 | 0.06 | 0.07 | 0.06 | 0.07 | 0.06 | 0.07 | 0.06 | 0.09 | 0.07 | 0.08 | 0.06 |
| **W** | 0.01 | 0.02 | 0.02 | 0.01 | 0.05 | 0.02 | 0.01 | 0.01 | 0.01 | 0.01 | 0.01 | 0.05 | 0.02 | 0.01 |
| **Y** | 0.03 | 0.06 | 0.05 | 0.03 | 0.04 | 0.06 | 0.05 | 0.05 | 0.04 | 0.04 | 0.03 | 0.04 | 0.03 | 0.04 |
| **AA** | M | H | Y | H | W | Y | Y | Y | F | H | I | W | I | F |
| **Entropy** | 0.96 | 0.98 | 0.97 | 0.97 | 0.98 | 0.97 | 0.97 | 0.97 | 0.97 | 0.97 | 0.95 | 0.98 | 0.96 | 0.97 |
| **Wt** | 0.05 | 0.04 | 0.05 | 0.04 | 0.05 | 0.06 | 0.05 | 0.05 | 0.07 | 0.05 | 0.1 | 0.05 | 0.09 | 0.07 |
| **Max** | 0.13 | 0.09 | 0.1 | 0.08 | 0.09 | 0.1 | 0.09 | 0.09 | 0.1 | 0.08 | 0.14 | 0.08 | 0.12 | 0.1 |
| **Max_aa_ gh19** | L | L | L | L | L | L | A | L | A | L | L | L | L | L |
| **Diff** | 0.08 | 0.05 | 0.05 | 0.04 | 0.04 | 0.04 | 0.04 | 0.04 | 0.03 | 0.03 | 0.03 | 0.03 | 0.03 | 0.03 |
